# Individual differences in solving arithmetic word problems

**DOI:** 10.1186/1744-9081-9-28

**Published:** 2013-07-24

**Authors:** Sabrina Zarnhofer, Verena Braunstein, Franz Ebner, Karl Koschutnig, Christa Neuper, Manuel Ninaus, Gernot Reishofer, Anja Ischebeck

**Affiliations:** 1 Cognitive Psychology and Neuroscience, Department of Psychology, University of Graz, Universitaetsplatz 2 / III, 8010, Graz, Austria; 2 Department of Radiology, Medical University of Graz, Auenbruggerplatz 9, 8036, Graz, Austria; 3 Department of Knowledge Discovery, University of Technology of Graz, Krenngasse 37/ IV, 8010, Graz, Austria

**Keywords:** fMRI, Cognitive styles, Number processing, Visual cortex, Angular gyrus

## Abstract

**Background:**

With the present functional magnetic resonance imaging (fMRI) study at 3 T, we investigated the neural correlates of visualization and verbalization during arithmetic word problem solving. In the domain of arithmetic, visualization might mean to visualize numbers and (intermediate) results while calculating, and verbalization might mean that numbers and (intermediate) results are verbally repeated during calculation. If the brain areas involved in number processing are domain-specific as assumed, that is, that the left angular gyrus (AG) shows an affinity to the verbal domain, and that the left and right intraparietal sulcus (IPS) shows an affinity to the visual domain, the activation of these areas should show a dependency on an individual’s cognitive style.

**Methods:**

36 healthy young adults participated in the fMRI study. The participants habitual use of visualization and verbalization during solving arithmetic word problems was assessed with a short self-report assessment. During the fMRI measurement, arithmetic word problems that had to be solved by the participants were presented in an event-related design.

**Results:**

We found that visualizers showed greater brain activation in brain areas involved in visual processing, and that verbalizers showed greater brain activation within the left angular gyrus.

**Conclusions:**

Our results indicate that cognitive styles or preferences play an important role in understanding brain activation. Our results confirm, that strong visualizers use mental imagery more strongly than weak visualizers during calculation. Moreover, our results suggest that the left AG shows a specific affinity to the verbal domain and subserves number processing in a modality-specific way.

## Background

It seems that inner speech and visual imagery fulfill an important role in our cognition. Nevertheless, it is unclear what influence they have on performance and brain activity during problem solving. Inner speech or “*thinking in words*” [1] as well as visual imagery or “*seeing with the mind*’*s eye*” [2] is used in a wide range of mental activities, ranging from memory to reasoning, and thus plays a key role in information processing [3].

The concept of the visual and verbal cognitive style links visual imagery with inner speech. A cognitive style is assumed to be a relatively stable characteristic that describes how an individual processes information, that is, how an individual thinks, perceives, and remembers [4,5]. Verbalizers report to repeat information during thinking verbally, whereas visualizers claim to represent information during thinking pictorially or schematically. The earliest model of the visual-verbal cognitive style was one-dimensional, with visualization and verbalization as two contrasting poles. In this model an individual may be classified as a visualizer or as a verbalizer e.g., [4,6]. However, on the basis of the current knowledge about the brain, suggesting that the visual processing system and the verbal processing system are anatomically and functionally independent, this model seems to be outdated. Blazhenkova and Kozhevnikov [7] therefore suggested a basic independence of the visual and verbal cognitive style during information processing. An individual might use visualization as well as verbalization in information processing. Depending on the context, however, he or she might show a preference for one of the two cognitive styles.

In the domain of arithmetic, a visual style might mean to visualize numbers and (intermediate) results while calculating or to move mentally along the mental number line. A verbal style, on the other hand, might mean that numbers and (intermediate) results are verbally repeated during calculation.

### Number processing

The intraparietal sulcus (IPS), bilaterally, and the left angular gyrus (AG) are proposed to be involved in the processing of numbers [8]. Quantity is proposed to be processed in the left and right horizontal segment of the IPS. Numbers are thought to be ordered from small to large on a mental number line [9]. This representation subserves semantic knowledge about numerical quantities, for example, that two is smaller than nine. The left AG is assumed to subserve the long term memory retrieval for arithmetic fact knowledge (e.g., multiplication tables) [8].

Some researchers additionally proposed that the left and right IPS as well as the left AG show some modality specificity. First, it is proposed that the IPS has an affinity to the visual domain as the IPS also plays a role in visual attention [10-12], and in visual-spatial short-term memory [13,14]. Second, it is proposed that the left AG has an affinity to the verbal domain as it is assumed to support the arithmetic fact retrieval from verbal long-term memory [8].

It is an open question if mental arithmetic is influenced by language processing. On the one hand some theories propose that language processing has no influence on mental arithmetic e.g., [15,16] or that language exerts influence only at a peripheral encoding stage. Other theories, on the other hand, propose that language representations have an influence at more central processing stages of mental arithmetic [17-21]. Dehaene and Cohen proposed a triple-code model [17-21]. While some aspects of number processing might be independent of language, other aspects might be dependent on the form of presentation of a problem. In the triple-code model, three codes of number representations are proposed, the Arabic code, the quantity or magnitude code, and the verbal code. The visual Arabic code is proposed to be localized in the left and right inferior ventral occipitotemporal areas, where numbers are thought to be represented as strings of digits. This representation subserves, for example, multidigit operations. The analogical quantity or magnitude code is proposed to be localized in the left and right horizontal segment of the IPS, and the verbal code (number words) within the left AG.

Noël and Seron [22] developed a “preferred entry code” hypothesis. This hypothesis assumes a preferred entry to access number knowledge and calculation procedures. While some individuals might have access to number knowledge from Arabic representations, other individuals might have access to number knowledge from verbal representations. If a number is presented in the non-preferred modality it is transcoded to the preferred modality from which magnitude and other semantic number knowledge is accessible. In general, it is an open question if number representation relies on modality-specific processes [17-24] or if all numerical input is gated to an amodal representation [15,16].

A meta-analysis showed that the ability to process numbers and perform calculations relies on a large number of brain regions [25]. In addition to the brain areas mentioned above, activation during number and calculation tasks were also observed in the cingulate gyri, the insula, and the cerebellum. Furthermore, activation in dorsolateral and frontopolar areas of the prefrontal cortices was modulated by task difficulty [25].

It is an open question how individual differences in arithmetic problem solving are reflected in the brain. In particular, are there differences in brain activation due to the cognitive style of an individual? An fMRI study by Burbaud and colleagues [26] using an arithmetic task found brain activation differences between visualizers and verbalizers in areas involved in verbal and visual processing, but not in brain areas associated with number processing.

In a previous study [27] we found that the higher the self reported tendency to verbalize during mental arithmetic the higher the activation in brain areas related to language and auditory processing. It is unclear, why we found significant correlations only for the verbalizer dimension but not for the visualizer dimension. We think that the basic arithmetic problems (subtraction and multiplication problems) we used in the previous study were not ideal to study the visual cognitive style perhaps because the format of the basic arithmetic problems did not support visualization (i.e. only the numbers and intermediate results could be visualized and no scenes, colors etc.). For the present study, arithmetic word problems were used as stimuli instead of basic arithmetic problems because we expected that arithmetic word problems would offer more flexibility for visualization and verbalization. The solving of arithmetic word problems requires more steps than the solving of basic arithmetic problems. The arithmetic word problem must be encoded and then translated into a more abstract representation, a basic arithmetic problem, which then has to be solved. Referring to the triple-code model [17-21], encoding and translating might depend on language processes while the influence of language processes on the solving of the basic arithmetic problems might depend on characteristics of the problem.

### Arithmetic word problems

The solving of arithmetic word problems makes high demands on cognition. Behavioral studies showed that students reported to visualize when solving difficult or novel arithmetic word problems, and to verbalize when solving less difficult arithmetic word problems [28]. Mental visual representations could be classified as primarily schematic or primarily pictorial. Use of schematic spatial representations is associated with success in mathematical problem solving, whereas use of pictorial representations is negatively correlated with success [29]. Moreover use of visual images is positively correlated with higher arithmetic word problem solving performance [30].

### The present study

If an individual’s cognitive style reflects a general preference for a stimulus modality, we hypothesized, first, that strong verbalizers show higher activation in brain areas associated with language and auditory processing than weak verbalizers, and that strong visualizers show higher activation in brain areas associated with visual processing than weak visualizers while solving arithmetic word problems.

If the brain areas involved in number processing are modality-specific, that is, if the left AG shows an affinity to the verbal domain, and if the left and right IPS shows an affinity to the visual domain, the activation of these areas could show a dependency on an individual’s cognitive style. We hypothesized, second, that strong verbalizers might show higher activation within the left AG than weak verbalizers, and that strong visualizers might show higher activation within the IPS than weak visualizers.

In the fMRI session, arithmetic word problems were presented in randomized order in an event-related design (e.g., ‘Anna goes for a walk. She walks 4 kilometers per hour. What distance does she cover in 3 hours?’). The individual’s habitual use of visualization and verbalization during mental arithmetic was assessed with a short self-report assessment.

## Methods

### Participants

45 healthy young adults (27 female) participated in the fMRI study. All were native speakers of German, right-handed, all had normal or corrected to normal vision, and no history of neurological or psychiatric illness. All participants were university students. The participants had an average age of 24 years (*SD* = 5.53). The study was approved by the ethics committee of the Medical University Graz.

Nine participants (four female) had to be excluded from the analysis. Seven participants (four female) had to be excluded because of technical malfunction and two participants had to be excluded because of excessive motion. The behavioural and functional data were analyzed for the remaining 36 participants (23 female; age: *M* = 23.72, *SD* = 4.39).

### Self-assessment of visualization and verbalization in solving arithmetic word problems

We designed a short self-report measure to assess the use of verbalization and visualization in solving arithmetic word problems. Four arithmetic word problems were presented in written form to each participant (e.g., Anna buys a book for 29€ and a DVD. She pays 44€. How expensive was the DVD?). Each of the four arithmetic word problems contained one of the four arithmetic operations (addition, division, multiplication, subtraction). Subsequent to each of the four arithmetic word problems, three statements about visualizing (e.g., If I have to solve this problem, I imagine Anna, the book, and/ or the DVD) and two statements about verbalizing (e.g., If I have to solve this problem I repeat the problem and the (intermediate) results in my mind and hear the sound of my own voice.) were presented in written form. The participant had to assess his or her behavior on all items on a five point rating scale. Altogether, there were twelve statements assigned to visualizing in solving arithmetic word problems, and eight statements were assigned to verbalizing in solving arithmetic word problems. The values of the twelve statements of visualization were added and the sum was divided by twelve to create a mean score of visualization in solving arithmetic word problems for each participant (maximum range of values: *R* = 1 – 5). The values of the eight statements of verbalization were added and the sum was divided by eight to create a mean score of verbalization in solving arithmetic word problems for each participant (maximum range of values: *R* = 1 – 5).

### Intelligence test

The intelligence test was conducted to assess the relationship between cognitive style and intelligence to test whether the assessed use of visualization and verbalization during solving arithmetic word problems is dependent on intelligence. We used a standardized intelligence test [Intelligence Structure Test in German language: I-S-T 2000 R; [31]. The battery contains a basic module measuring verbal, numerical, and figural intelligence. Each content area of intelligence is assessed through three subtests.

### Stimuli

For the fMRI session, arithmetic word problems in German were constructed out of 15 problems per operation (addition, division, multiplication, subtraction). Additions consisted of problems with two one-digit addends and one- or two-digit solutions. Problems with two identical summands (e.g., 4+4) were not presented. Divisions were chosen from the multiplication table. All were two-digit dividends divided by an one-digit divisor problems with one-digit solutions. The one-digit divisors were 3, 4, 6, 7, 8, and 9. Multiplications were also chosen from the multiplication table. All were one-digit times one-digit problems with two-digit solutions. The one-digit operands were 3, 4, 6, 7, 8, and 9. Problems with other operands (1, 2 or 5) and ties (two equal operands, e.g., 4×4) were not presented [32]. Subtractions were chosen of problems with one- or two-digit minuends minus a one-digit subtrahend with a one-digit solution. Each problem was presented three times. The problem was twice part of a short story (STORY; e.g., Anna goes for a walk. She walks 4 kilometers per hour. What distance does she cover in 3 hours?), and once the arithmetical problem was formulated (FORMULATION; e.g., What result do you obtain when multiplying 3 by a factor of 4?). The motivation for using these two conditions was that each condition may support a specific cognitive style. Visualizers may visualize the content of an arithmetic word problem as well as the numbers and (intermediate) results. As a consequence, they might visualize more in the condition STORY. Verbalizers, on the other hand, may repeat the problem and (intermediate) results verbally. As a consequence, they might verbalize equally in both conditions. Distractors for the multiplication problems were operand-related, that is, i.e. solutions of related problems. For example, the distractors of the problem 3×4 would be 8 (2×4), 16 (4×4), 9 (3×3), and 15 (3×5). To prevent the use of shortcut strategies, distractors for the addition, division, and subtraction problems were chosen such that the differences to the correct solution (±1 or ±2) were balanced over all problems. All problems were intermixed and presented in randomized order. The fMRI session consisted of 180 trials in total.

### Procedure

Before entering the scanner, the participant had to fill in the short self-assessment questionnaire of visualization and verbalization in solving arithmetic word problems.

**Figure 1:**
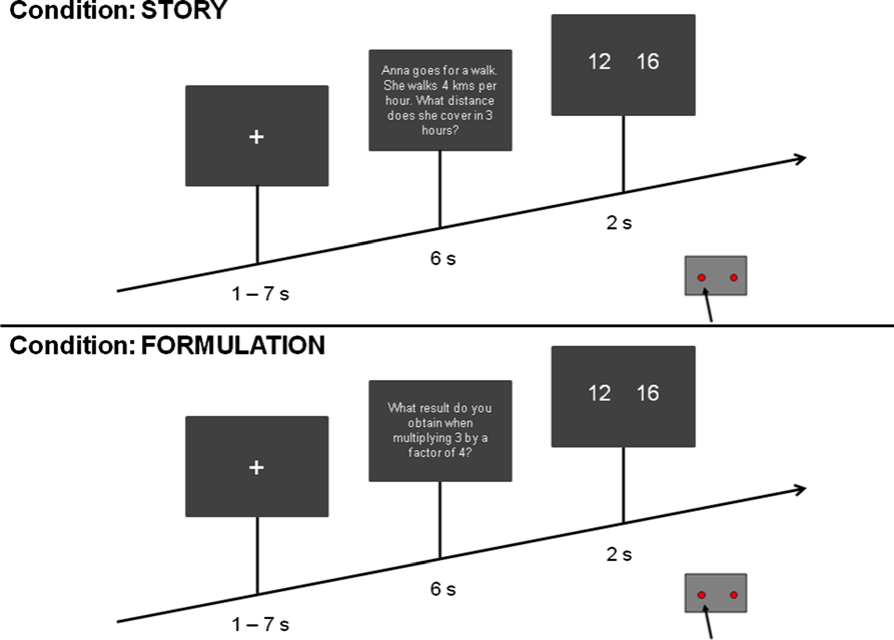
**Schematic of the task.** Arithmetic word problems were presented in German language in randomized order (180 trials). Participants had to choose the alternative that corresponds the solution. Response times were measured from the onset of the presentation of the alternatives.

During the fMRI session, the participant had to solve 180 arithmetic word problems presented in randomized order. Each trial started with the presentation of a fixation cross for 1 to 7 seconds (s; average presentation time of 4 s), followed by the presentation of the problem for 6 s. Then the two alternatives - the solution and one of the distractors – were presented for 2 s, giving a total trial duration of 9 to 15 s. The scheme of the task is shown in Figure 1. Response times were measured from the onset of the presentation of the two alternatives. The participant had to indicate on which side of the screen the correct response was presented by pressing the corresponding button with his or her right hand. All stimuli were presented in white letters against a grey background. The functional session lasted approximately 30 minutes.

Stimulus presentation, synchronization with the scanner, and response recording were programmed and controlled by the software Presentation (Neurobehavioral Systems).

### fMRI data acquisition

Imaging was performed with a 3 T Siemens Magnetom Tim Trio scanner (Siemens Medical Solutions, Erlangen, Germany) and a 32-channel head coil. For the anatomical images, an isotropic MPRAGE sequence was used (TR = 1900 ms, TE = 2.19 ms, TI = 900 ms, flip angel = 9° inplane acquisition matrix = 256×256, FoV = 256 (saggital view), 176 partitions, slice thickness 1 mm). For the functional images, a T2*-weighted EPI-sequence was employed (TR/TE = 2000 ms/ 24 ms, matrix = 64×64, FoV = 192, 31 axial contiguous slices covering the whole brain, inplane resolution: 3×3 mm, slice thickness 3 mm and 0.90 mm gap, acquired descendingly and parallel to the AC-PC line) sensitive to brain oxygen-level dependent (BOLD) contrast. Participants wore ear plugs as protection against the scanner noise.

### fMRI data analysis

Data analyses and pre-processing were performed with the SPM software (SPM 8, Wellcome Department of Cognitive Neurology, London, U.K.). The first four functional images of each participant were discarded to ensure signal stabilization. The remaining functional images were motion-corrected, unwarped, and corrected for slice acquisition time. The functional images were then normalized to the MNI anatomical template. Images were finally smoothed with a Gaussian kernel of 8 mm FWHM.

Statistical analyses were performed on the basis of the general linear model implemented in SPM8. A model with two conditions was analyzed (formulation, story). The experiment was analyzed on the basis of single events (event-related). The trial onsets of the single events were calculated from the logfiles saved with the software Presentation (Neurobehavioral Systems) for each participant separately. The delta-function of the trial onsets for each condition was convolved with the canonical form of the hemodynamic response function and its first and second temporal derivative. A high-pass filter of 1/200 Hz and an autocorrelation model (AR(1)) were employed, but no low-pass filter and no global normalization. The values of the use of verbalization as well as visualization in solving arithmetic word problems (assessed with a short self-assessment) were included as covariates of interest, and verbal intelligence was included as a regressor of no interest in a whole brain analysis (multiple regression). Significant activation clusters were determined using an uncorrected voxelwise *p* < .005 level, and a family-wise error (FWE) correction for multiple comparisons at *p* < .05 on cluster level.

### Behavioural data analysis

Rates of correct responses were arcsin√p transformed to achieve approximate variance equality [33].

Two separate linear regression analyses were performed. The values of the use of verbalization as well as visualization in solving arithmetic word problems (criterion variables) were regressed on verbal intelligence, numerical intelligence, figural intelligence, response times and arcsin√p transformed rates of correct responses for both conditions (formulation, story; predictor variables).

Response times and the arcsin√p transformed rates of correct responses were entered into two general linear models for repeated measures with the factors WORD PROBLEM (formulation, story), and OPERATION (addition, division, multiplication, subtraction).

## Results

### Cognitive style

The self-reported use of verbalization (*M* = 3.31, *S*.*D*. = .95) and visualization (*M* = 2.66, *S*.*D*. = .81) did not correlate (*r* = −.297, *p* = 0.08, n.s.; *n* = 36).

We observed no significant correlations between the self-reported use of verbalization or visualization during solving arithmetic word problems and verbal, numerical or figural intelligence as well as performance in the arithmetic task (Verbalization: *R*^*2*^ = .298, *F*_7,27_ = 1.639, *p* = .167, n.s.; *n* = 36; Visualization: *R*^*2*^ = .112, *F*_7,27_ = .487, *p* = .835, n.s.; *n* = 36). This indicates, first, that an individual’s self-reported habitual use of verbalization and visualization during solving arithmetic word problems does not depend on intelligence. Second, an individual’s self-reported habitual use of verbalization and visualization during solving arithmetic word problems has no influence on mathematical achievement. Statistics for the regression coefficients are presented in Table 1.

**Table 1:** Statistics for the regression coefficients

	**Verbalization**				**Visualization**			
***Coefficients***	***B***	***Beta***	***t***	***p***	***B***	***Beta***	***t***	***p***
*Verbal IQ*	-.314	-.327	1.703	.10	.018	.016	.073	.94
*Numerical IQ*	-.263	-.397	−1.519	.14	.095	.119	.406	.69
*Figural IQ*	.175	.179	.702	.49	-.334	-.283	-.986	.33
*RT formulation*	.007	.105	.221	.83	-.021	-.270	-.504	.62
*RT story*	-.033	-.510	−1.084	.29	.041	.529	1.000	.33
*C formulation*	.885	.088	.386	.70	1.341	.111	.431	.67
*C story*	−1.523	-.217	-.983	.33	2.096	.248	.998	.33

Note: RT formulation … response times for word problems of the condition formulation, RT story … response times for word problems of the condition story, C formulation … arcsin√p transformed rate of correct responses for word problems of the condition formulation, C story … arcsin√p transformed rate of correct responses for word problems of the condition story.

The table shows the statistics for the regression coefficients of two separate linear regressions. The criterion variables were the values of the use of verbalization as well as visualization in solving arithmetic word problems (assessed with a short self-assessment). The predictor variables were verbal intelligence, numerical intelligence, figural intelligence, response times and arcsin√p transformed rates of correct responses for both conditions (formulation, story). The criterion variables were regressed on the predictor variables in two separate analyses. No significant correlation between the self-reported use of verbalization or visualization during solving arithmetic word problems and verbal, numerical or figural intelligence as well as performance was observed.

However, an additional correlation matrix between cognitive style and intelligence showed that verbalization and verbal intelligence were significantly negatively correlated. It should be mentioned that this correlation is not significant any more after Bonferroni correction. No significant correlations between the self-reported use of verbalization or visualization in solving arithmetic word problems and numerical or figural intelligence were observed (*n* = 36), and no significant correlation between the self-reported use of visualization in solving arithmetic word problems and verbal intelligence was observed. Statistics for the correlations between cognitive style and intelligence are presented in Table 2.

**Table 2:** **Correlations between verbalization**/ **visualization and intelligence**

	**Verbal intelligence**	**Numerical intelligence**	**Figural intelligence**
Visualization	.022 (*p* = .900)	-.030 (*p* = .862)	-.140 (*p* = .424)
Verbalization	-.345 (*p* = .042)*	-.293 (*p* = .087)	-.160 (*p* = .358)

The table shows the product moment correlation coefficients between verbalization/ visualization and intelligence, statistical significance is given in brackets. No significant correlations between the self-reported use of verbalization or visualization in solving arithmetic word problems and numerical or figural intelligence were observed (*n* = 36). Moreover, no significant correlation between the self-reported use of visualization in solving arithmetic word problems and verbal intelligence was observed (*n* = 36), but a significant negative correlation between the self-reported use of verbalization in solving arithmetic word problems and verbal intelligence was observed (*n* = 36). However, it should be mentioned that this correlation is not significant any more after Bonferroni correction. (We used a Bonferroni correction for the number of content areas of intelligence for each modality. Thus, the critical *p* value was .017 (.05 / 3)).

The internal consistency (Cronbach’s Alpha) for the self-reported use of verbalization is .87 (8 items, corrected item-scale-correlations vary between .43 and .83) and for the self-reported use of visualization .88 (8 items, corrected item-scale-correlations vary between .25 and .79). For both scales, the internal consistencies are good, so that they can be used as covariates in a general linear model [34,35].

### Response times and rates of correct responses

Formulated word problems were solved faster than word problems that are part of a short story (737 ms vs. 760 ms; *F*_1,35_ = 8.83, *p* < .01). Additions, divisions, and subtractions were solved faster than multiplications, and additions were solved faster than divisions (RT_add_ = 705 ms, RT_div_ = 747 ms, RT_mult_ = 822 ms, RT_subt_ = 720 ms; *F*_3,33_ = 26.52, *p* < .001). The interaction between word problem and operation was not significant (*F*_3,33_ = 1.42, *p* = .26, n.s.).

No difference was observed for the rates of correct responses between formulated word problems and problems that are part of a short story (94.95% vs. 95.79%; *t*_35_ = −.381, *p* = .71, n.s.). Additions were solved more accurately than divisions and multiplications, and subtractions were solved more accurately than multiplications (C_add_ = 97.08%, C_div_ = 95.19%, C_mult_ = 93.84%, C_subt_ = 95.46%; *F*_3,33_ = 9.01, *p* < 0.001). The interaction between word problem and operation was not significant (*F*_3,33_ = 2.33, *p* = .09, n.s.).

### Whole brain analyses

**Figure 2:**
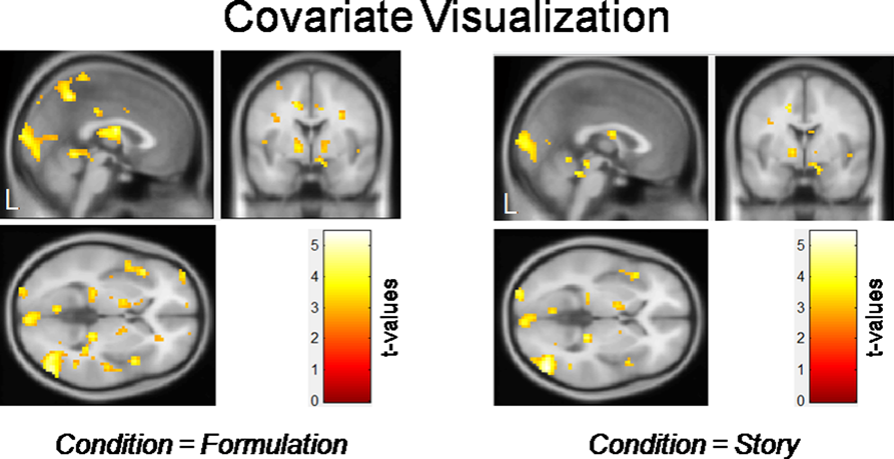
**Whole brain analysis: visualization.** The values of the use of verbalization as well as visualization in solving arithmetic word problems (assessed with a short self-assessment) were included as covariates in a whole brain analysis (multiple regression). The value of visualization was included as a covariate of interest and the value of verbalization was included as a predictor of no interest (*p* < 0.005).

Significant correlations were observed in the whole brain analysis between brain activation and the mean score of visualization. For both conditions, the higher the score of visualization the higher the activation in the left and right superior occipital gyrus, the left and right middle occipital gyrus, the right inferior occipital gyrus, the left and right lingual gyrus, the left and right calcarine gyrus, the left and right cuneus, the right superior temporal gyrus, the right middle temporal gyrus, the right inferior temporal gyrus; the right fusiform gyrus, and the left and right thalamus (see Table 3 and Figure 2).

**Table 3:** **Whole brain analysis**: **visualization**

**Condition**	**Side**	**Area**	**x**	**y**	**z**	**k**	**Z**
Story	left	superior occipital gyrus	−12	−85	19	589^*1^	4.40
Story	right	middle temporal gyrus	51	−73	1	539^*2^	4.34
Formulation	left	lingual gyrus	−6	−67	7	2282^*3^	4.54

Note: Coordinates are reported as given by SPM8 (MNI space) and correspond only approximately to Talairach and Tournoux space (Brett et al., [60], Talairach and Tournoux, [61]). The label denotes the location of the maximum.

^*1^ left calcarine gyrus; left superior occipital gyrus; left cuneus; right cuneus; left superior occipital gyrus; right calcarine gyrus; left lingual gyrus; right middle occipital gyrus; left middle occipital gyrus.

^*2^ right inferior occipital gyrus; right middle temporal gyrus; right middle occipital gyrus; right inferior temporal gyrus; right fusiform gyrus; right lingual gyrus

^*3^ left calcarine gyrus; right middle temporal gyrus; left cuneus; right inferior occipital gyrus; right superior occipital gyrus; right middle occipital gyrus; right cuneus; right inferior temporal gyrus; right thalamus; right superior temporal gyrus; left superior occipital gyrus; right fusiform gyrus; left thalamus; right calcarine gyrus; left lingual gyrus; right lingual gyrus.

The values of the use of visualization in solving arithmetic word problems (assessed with a short self-assessment) were included as a covariate in the whole brain analysis. (*Abbreviations*: *k* cluster size, *Z Z*-value; activation significant at *p* < 0.005 uncorrected and *p* < 0.05 FWE corrected on cluster level).

**Figure 3:**
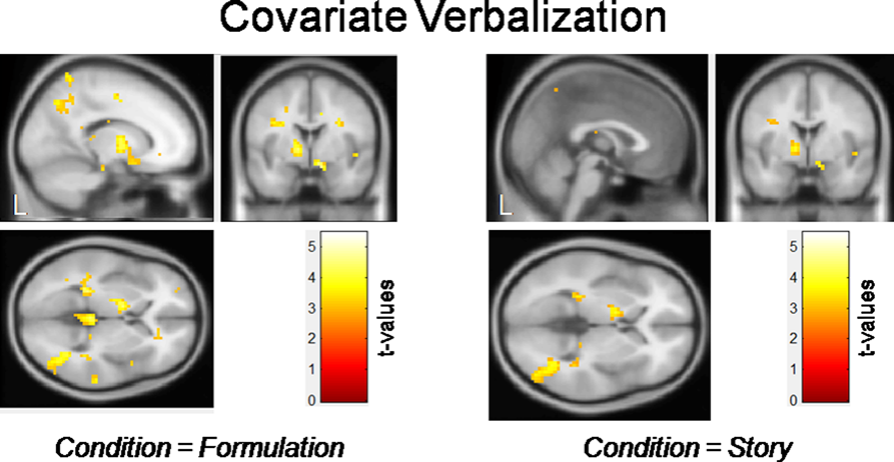
**Whole brain analysis: verbalization.** The values of the use of verbalization as well as visualization in solving arithmetic word problems (assessed with a short self-assessment) were included as covariates in a whole brain analysis (multiple regression). The value of verbalization was included as a covariate of interest and the value of visualization was included as a predictor of no interest (*p* < 0.005).

Significant correlations were also observed in the whole brain analysis between brain activation and the score of verbalization. The higher the score of verbalization the higher the activation within the left angular gyrus, the left hippocampus, the right thalamus, the left precuneus, and the right pallidum for the condition FORMULATION. For the condition STORY a significant activation within the right middle temporal gyrus was observed (see Table 4 and Figure 3).

**Table 4:** **Whole brain analysis**: **verbalization**

**Condition**	**Side**	**Area**	**x**	**y**	**z**	**k**	**Z**
Story	right	middle temporal gyrus	36	−58	−5	432	4.53
Formulation	left	angular gyrus	−27	−37	4	254	4.27
Formulation	right	thalamus	36	−58	−5	257	4.26
Formulation	left	precuneus	−9	−55	67	291	4.06
Formulation	right	thalamus	27	−31	13	233	4.05
Formulation	right	pallidum	9	2	−14	230	3.95

Note: Coordinates are reported as given by SPM8 (MNI space) and correspond only approximately to Talairach and Tournoux space (Brett et al., [60], Talairach and Tournoux, [61]). The label denotes the location of the maximum.

The values of the use of verbalization in solving arithmetic word problems (assessed with a short self-assessment) were included as a covariate in the whole brain analysis. (*Abbreviations*: *k* cluster size, *Z Z*-value; activation significant at *p* < 0.005 uncorrected and *p* < 0.05 FWE corrected on cluster level).

No significant results were observed in the whole brain analyses between brain activation and verbal intelligence.

## Discussion

The aim of the present fMRI study was to investigate the influence of visualization and verbalization on cerebral activation patterns while solving arithmetic word problems. The habitual use of visualization and verbalization during solving arithmetic word problems of each of the 45 right-handed participants was assessed with a short self-report measure. In the functional session, 180 arithmetic word problems that had to be solved by the participants were presented in an event-related design. With regard to visualization, we found that the higher the score of visualization the higher the activation in brain areas related to visual processing. With regard to verbalization, we found that the higher the score of verbalization the higher the activation in the left angular gyrus.

Visualization was associated with higher activation within brain areas related to visual processing, namely the left and right superior occipital gyrus, the left and right middle occipital gyrus, the right inferior occipital gyrus, the left and right lingual gyrus, the left and right calcarine gyrus, the left and right cuneus, the right superior temporal gyrus, the right middle temporal gyrus, the right inferior temporal gyrus, the right fusiform gyrus, and the left and right thalamus. This suggests that visualizers visualize the content or the numbers of the presented arithmetic word problem during calculation. The calcarine sulci belong to the primary visual cortex in the occipital lobe of the human brain, the first cortical structure to process incoming visual information e.g., [36,37]. The lingual gyrus, bilaterally, is a ventral stream visual brain region that is part of the ‘what’ system [38] activated during processing information presented in a visual form [39,40], object processing [41,42], and visuo-spatial operations [43]. In a previous study Hsu and colleagues [44] also found that brain activation within the left lingual gyrus was significantly correlated with visualization. The primary visual cortex around the calcarine fissure can be activated during both visual perception and visual recall [36]. The cuneus is located in the tertiary visual cortex and modifies information transferred via primary visual cortex to extrastriate cortices [45]. The left and right fusiform gyrus is located in the extrastriate cortex [39] and involved in the perception of objects such as faces e.g., [46-49]. Neuroimaging studies already showed that object concepts of different categories (e.g., animals or tools) are represented in partially distinct neural networks within the extrastriate cortex [50]. Neuropsychological data suggest the existence of two distinct imagery subsystems that encode and process visual information in different ways, that is an object imagery system and a spatial imagery system. The object imagery system (ventral pathway to the inferior–temporal cortex) processes the visual appearance of objects and scenes in terms of their shape, color information, and texture [38]. A high object-processing ability is associated with a more efficient use of visual-object resources, resulting in less neural activity in the object-processing pathway [51]. The spatial imagery system (dorsal pathway to the posterior parietal cortex) processes object location, movement, spatial relationships and transformations, and other spatial attributes of processing [38]. The use of schematic spatial representations is positively correlated with success in mathematical problem solving, whereas the use of pictorial representations is negatively correlated with success [29].

Although we found significant brain activation as a function of visualization in brain areas related to visual processing, we observed no modulation of activation in the left and right IPS as a function of the self-reported tendency to use visualization while solving arithmetic word problems. Some researchers have proposed that the IPS shows some modality-specificity in number processing as the IPS also plays a role in visual attention [10-12], and in visual-spatial short-term memory [13,14]. Other researchers, however, have proposed that the IPS hosts an amodal representation of quantity [16,19]. These numerical representations are thought to be ordered from small to large on a mental number line which has been proposed to be represented in the IPS [16,19]. In the present study, the activation of the left and right IPS was not modulated by visualization. It could be concluded that the left and right IPS shows no specific affinity to the visual domain and subserves number processing in a domain-general way. This interpretation corresponds to the proposals of the triple-code model [17-21]. Some previous studies dealt with the functional organization of the IPS. Simon and colleagues [52], for example, localized calculation and language-related activations within the IPS where they overlapped with visuospatial activations. The authors found two distinct activations. One activation was unique to calculation in the bilateral anterior IPS mesial to the supramarginal gyrus. The other activation was shared with phoneme detection in the left IPS mesial to the left AG. This finding corresponds to the triple-code model [17-21] in which different mental representations of numbers are proposed. The analogical quantity or magnitude code is proposed to be localized in the left and right horizontal segment of the IPS, and the verbal code is proposed to be localized within the left AG [17-21]. Knops and colleagues [53] found that parts of the superior parietal lobule, which were activated during eye movements, specifically predicted addition versus subtraction processes, whether performed with Arabic digits or with sets of dots. This finding is consistent with the suggestion of a spatial coding of numbers which might be represented on a mental number line.

A higher degree of verbalization was accompanied by a higher activation within the left AG, the right middle temporal gyrus, the right thalamus, the left precuneus, and the right pallidum. The left AG is assumed to support the long term memory retrieval for arithmetic fact knowledge [8]. Arithmetic fact knowledge is required, for example, in the skilled solving of multiplication problems by retrieving the result from verbal long-term memory, namely, from the multiplication tables learned in childhood. The left AG shows stronger activation for solving arithmetic problems for which participants report fact retrieval whereas the application of procedural strategies is accompanied by activation in a fronto-parietal network [54]. These findings link the left AG to arithmetic fact retrieval. In the present study, the activation of the left AG was modulated by verbalization. It might therefore be concluded that the left AG shows a specific affinity to the verbal domain. In a previous study [27], however, we found no modulation of activation in the left AG as a function of verbalization. Therefore, more studies are needed to answer the question whether the left AG is involved in number processing in a modality specific way. Nevertheless, our findings could be interpreted to support the proposals of the triple-code model [8]. Our results are consistent with the idea that the IPS is involved in number processing in a domain-general way and that the left AG is involved in number processing in a modality-specific way.

A recently published fMRI study by Kraemer and colleagues [55] observed that individuals tend to mentally convert information that is presented in a not preferred mode to the preferred mode of processing. The authors observed that during reading, visualizers showed activation in the right fusiform gyrus, an area implicated in visual processing, whereas during picture presentation, verbalizers showed activation in the left supramarginal gyrus, an area implicated in verbal processing. These findings indicate that individuals with a visual cognitive style could have a tendency to convert linguistically presented information into a visual mental representation. Similarly, individuals with a verbal cognitive style could have a tendency to convert pictorially presented information into a verbal mental representation. Kraemer and colleagues [55] also found similar activity for visualizers and verbalizers as we found in our study.

It is unclear why we did not find any significant activation in brain areas that are involved in verbal and auditory processing as a function of verbalization. In a previous study [27], we found that the higher the self-reported tendency to verbalize the higher the activation in areas related to verbal (left and right supramarginal gyrus) and auditory processing (left and right Heschl’s gyrus, left and right Rolandic Operculum. It is possible that, given the arithmetic word problems, verbalization was present already to a high degree, so that individual differences no longer played a role. As a consequence, activation in verbal brain areas did not show any modulation due to verbalization.

Some researchers proposed that we represent and retrieve knowledge together with the sensory and motor features that were activated during its acquisition [56]. Regarding number processing, several studies showed that participants make associations between number magnitude and space. Numbers seem to be ordered from left to right on a “mental number line” as participants classify small digits faster with the left hand and larger digits faster with the right hand (SNARC effect; for a review, see, [57]). A recently published fMRI study showed that the intraparietal sulcus, frontal eye fields, and supplementary motor areas are sensitive to the congruency between visually presented numerical and spatial intervals [58]. Moreover, brain activation within the motor cortex, the precentral gyrus, seems to be lateralized. Visually presented small numerals mainly activate left-lateral premotor cortical regions in participants who usually use their right hand to gesture small numbers”, and right-lateral premotor cortical areas in participants who usually use their left hand to gesture small numbers [59]. Our results with regard to visual and verbal cognitive styles also fit well into the idea of embodiment in numerical cognition, as our brain’s activation reflects our way of thinking even in a domain that is as abstract as arithmetic problem solving. Note, that the coordinates in the results section are reported as given by SPM8 (MNI space) and correspond only approximately to Talairach and Tournoux space [60,61]. For future studies it might be of interest, for example, if strong visualizers show a larger association between number magnitude and space than weak visualizers because they acquisit, store, and retrieve numbers in a visual-spatial way.

For future studies in this field, second, an additional recording of eye movements would be wise to clarify reading or decoding strategies in dependence on cognitive styles. A potential problem with the presented stimulus format, namely arithmetic word problems, is that different cognitive styles may lead to different reading or decoding strategies. Therefore, it is questionable if the observed effects are due to the execution of arithmetic strategies or due to the translation of the problems into a more abstract representation that can then be subjected to familiar arithmetic problem solving strategies. Third, the inclusion of a non-numerical control condition would be advantageous to exclude influences on brain activation that cannot be attributed to cognitive styles (e.g., reading speed, meta-cognition). A possible non-numerical control condition to arithmetic word problems may be reading. In a reading condition, a short text of the same length as the texts of the other conditions might be presented that does not contain an arithmetic problem. For the analyses, the activation of the reading condition could be subtracted from the activation of the other conditions.

## Conclusions

We tried to assess the visual-verbal cognitive style during solving arithmetic word problems with a short self-report measure. Our results indicate, first, that people who say they visualize more show more activity in brain areas related to visual processing during mental calculation, and second, that people who say they verbalize more show more activity in the left angular gyrus during mental calculation. Our results provide support for the proposal that visualizers see with their mind’s eye. Moreover our results provide support for the proposition that the left angular gyrus is involved in number processing in a modality-specific way.

## Competing interests

All authors declare not to have any conflict of interest including any financial, personal or other relationships with other people or organizations that could inappropriately influence, or be perceived to influence, their work.

## Authors’ contributions

The presented work was carried out in collaboration between all authors. All authors read and approved the final manuscript. SZ designed the experiment, collected and analyzed the data, interpreted the results, and wrote the paper. VB and MN helped at collecting the data and gave writing assistance. AI and CN made substantial contributions to the design, the acquisition of data, and the analysis and interpretation of data. FE, KK and GR provided technical help, and gave writing assistance at the method section.
